# EEG as a predictive biomarker of neurotoxicity in anti-CD19 CAR T-cell therapy

**DOI:** 10.1007/s00415-025-13102-3

**Published:** 2025-04-25

**Authors:** Federica Pondrelli, Lorenzo Muccioli, Federico Mason, Corrado Zenesini, Lorenzo Ferri, Gian Maria Asioli, Simone Rossi, Rita Rinaldi, Francesca Rondelli, Marianna Nicodemo, Roberto D’Angelo, Valentina Barone, Luisa Sambati, Umberto Pensato, Pier Luigi Zinzani, Beatrice Casadei, Francesca Bonifazi, Enrico Maffini, Elisabetta Pierucci, Pietro Cortelli, Paolo Tinuper, Francesca Bisulli, Maria Guarino

**Affiliations:** 1 IRCCS Istituto delle Scienze Neurologiche di Bologna, Bologna, Italy; 2 Department of Biomedical and Neuromotor Sciences, University of Bologna, Bologna, Italy; 3 Department of Information Engineering, University of Padova, Padova, Italy; 4 Department of Biomedical Sciences, Humanitas University, Pieve Emanuele, Milan Italy; 5 Department of Neurology, IRCCS Humanitas Research Hospital, Rozzano, Milan Italy; 6 IRCCS Azienda Ospedaliero-Universitaria di Bologna, Bologna, Italy; 7 Department of Medical and Surgical Sciences, University of Bologna, Bologna, Italy

**Keywords:** Chimeric antigen receptor (CAR) T-cell therapy, Lymphoma, Immune effector cell-associated neurotoxicity syndrome (ICANS), Biomarker, Electroencephalography (EEG), Quantitative analysis

## Abstract

**Objective:**

Immune effector cell-associated neurotoxicity syndrome (ICANS) is a potentially fatal complication of CD19-directed CAR T-cell therapy. The aim of this study was to investigate the role of EEG as a predictive biomarker of ICANS.

**Methods:**

In this prospective, monocentric, cohort study, consecutive refractory B-cell non-Hodgkin lymphoma patients undergoing CAR T-cell therapy had EEG assessments at fixed time points pre- and post-infusion. The risk of ICANS was evaluated according to EEG findings detected qualitatively, using a grading scale ranging from 0 (normal) to 3 (severely abnormal), and quantitatively, using power spectral and connectivity measures.

**Results:**

307 EEGs from 68 patients have been qualitatively evaluated, of whom 238 were eligible for quantitative analysis. Neurotoxicity manifested in 22/68 (32.4%) patients. Pre-infusion EEG abnormalities (grade 1 and 2) were qualitatively detected in 8/68 (11.7%) patients, emerging as a risk factor for ICANS [HR 5.8 (95%CI 2.6–12.9)]. Quantitative analysis of pre-infusion EEGs did not yield significative results. Post-infusion qualitative EEG abnormalities were associated to a higher risk of ICANS development [HR 11.6 (4.4–30.5) for grade 2; HR 9.7 (2.6–36.6) for grade 3]. Concerning the quantitative analysis, in post-infusion EEGs higher theta energy [HR 1.10 (1.03–1.16)] and delta + theta/alfa ratio [HR 1.37 (1.11–1.67)] were associated to higher risk of ICANS, while higher beta energy resulted protective [HR 0.91 (0.85–0.97)].

**Conclusions:**

Our study establishes EEG as a predictive tool for identifying patients at risk for ICANS before CAR T-cell infusion, who may benefit from prophylactic treatments, and anticipating ICANS onset following infusion, enabling early intervention.

**Supplementary Information:**

The online version contains supplementary material available at 10.1007/s00415-025-13102-3.

## Background

In recent years, anti-CD19 Chimeric Antigen Receptor (CAR) T-cell therapy has become a groundbreaking treatment for advanced B-cell non-Hodgkin lymphoma [1]. However, this procedure carries the risk of developing immune effector cell-associated neurotoxicity syndrome (ICANS) [2], with a reported incidence ranging from 37.5% to 77% [3] and a mortality rate of 3% [4]. Its pathogenesis appears to be at least partially linked to cytokine release syndrome (CRS), a systemic toxicity related to CAR T-cells infusion that almost invariably precedes ICANS onset. This inflammatory response contributes to blood–brain barrier (BBB) damage [4, 5] and subsequent transmigration of proinflammatory products into the central nervous system (CNS) [5]. Other factors increasing the risk of developing ICANS include elevated serum levels of C-reactive protein (CRP) and ferritin [6–11], lymphoma type, disease burden [2], and the specific CAR T-cell product [11].

A timely identification of neurotoxicity is essential for enabling prompt intervention with immunomodulatory treatments to prevent clinical deterioration [12]. However, the opportunity to prevent ICANS using prophylactic treatments is currently under investigation [13, 14] and preliminary evidence on the efficacy of anakinra is already available from non-randomized studies [13, 14]. In this context, predictive biomarkers would refine the selection of patients who may benefit from the prophylactic treatment. Nevertheless, the prediction of ICANS development remains, at present, a pressing unmet clinical need.

EEG is a widely available, non-invasive, and cost-effective technique, highly sensible in evaluating patients with encephalopathy, regardless of the aetiology [15], thus representing a candidate biomarker for ICANS. Studies investigating EEG changes during ICANS revealed significant alterations such as diffuse background slowing, sporadic rhythmic delta activity and epileptiform discharges [16–23], supporting its diagnostic role.

A previous study conducted at our Institute [24] describing neurological assessment and complications of a preliminary cohort of patients undergoing CAR T-cell therapy suggested that pre-infusion EEG abnormalities were associated with a higher risk of ICANS, in line with a recently published study [25]. Except these exploratory studies, there is a lack on EEG performed before CAR T-cell infusion and, even more notably, on EEGs performed following the infusion prior to the onset of ICANS, as EEGs are not routinely performed in this setting.

Therefore, the present study aims to shed light on the potential value of EEG as a predictive biomarker of ICANS in patients undergoing CAR T-cell therapy.

## Methods

### Study and cohort characteristics

This prospective, monocentric, real-life cohort study analysed the EEGs of adult patients with B-cell non-Hodgkin lymphoma who underwent CAR T-cell therapy at the Institute of Hematology “Seràgnoli” (Advanced Cell Therapy Program, IRCCS Azienda Ospedaliero-Universitaria di Bologna, Italy) between August 2019 and September 2022. All patients underwent a standardized neurological assessment protocol, as detailed in our previous works [24, 26], including serial EEG recordings at the following time points:

- Baseline: 15–20 days before the infusion day.

- T1; T3; T7; T14:1, 3, 7 and 14 days after the infusion day (referred to as T0, infusion day).

Additional EEGs were recorded depending on clinical concerns, such as suspicion or monitoring of neurotoxicity. ICANS grading was determined by the consulting neurologist based on the ICE score [2] and other clinical features (i.e. seizures, elevated intracranial pressure signs) as suggested by ASTCT Consensus [2].

Patients were administered prophylactic antiseizure medication (levetiracetam 1500 mg/die) starting 10–12 days before the infusion, in accordance with the CAR T-cell therapy-associated toxicity (CARTOX) working group recommendation [12].

We included patients with availability of: (1) at least the baseline EEG record, (2) neurologic examination and ICANS score at the time of EEG recording.

EEGs were excluded from analysis if excessive artifacts prevented their reliable evaluation.

The STROBE guidelines were used to ensure the reporting of this cohort study [27].

### Data collection

The following features of the included patients have been collected in an *ad hoc* database:

demographics, lymphoma characteristics, previous failed treatments, comorbidities, concomitant medications, laboratory and neuroimaging data, toxicities (CRS and ICANS) characteristics.

### EEG recording and evaluation

Routine EEGs (lasting at least 20 minutes) were recorded using the standard international 10–20 system for electrodes placement. A bipolar montage, 50 Hz notch filters and 0.5–40 Hz bandpass filters were applied. In the intensive care unit (ICU) setting, EEGs were recorded after the temporary discontinuation of anaesthetics. The included records were analysed qualitatively and quantitatively.

#### Qualitative evaluation

The records have been visually assessed independently by two expert neurophysiologists (FB, PT) who were blinded to patients’ histories and not involved in the neurological assessment of CAR T-cell therapy recipients.

While ACNS terminology classification [28] was used as a reference, some EEG features were evaluated differently to better describe the findings in CAR T-cell therapy recipients (routine EEG instead of long-term monitoring, non-intensive setting). An *ad hoc* EEG evaluation form was created, containing the following sections:

- Background activity (BA) (alternative options in square brackets)
- *Symmetry* [symmetric/mild asymmetry/marked asymmetry];
- *Predominant Background Frequency (PBF)* [beta/alpha/theta/delta];
- *Posterior Dominant Rhythm (PDR)* [present/absent];
- *Continuity* [continuous/nearly continuous/discontinuous/burst suppression];
- *Reactivity* [present/absent];
- *Voltage* [normal/attenuated/suppressed];
- *State changes (SC)* [absent/normal features/pathological features]:
- state changes are defined in ACNS Terminology as different types of background activities related to the level of alertness or stimulation [28]. In the present study, we defined state changes “pathological” if the posterior alpha rhythm was frequently replaced by long-lasting diffuse delta activity rapidly emerging after eye-closure, indicating somnolence (Example available in Supplemental Fig. 1). This pattern differs from physiological vigilance fluctuations characterized by gradual alpha rhythm disappearance while theta activity emerges, usually after few minutes in a relaxed state with closed eyes.
- Non-epileptiform abnormalities (NEA) [sporadic slowing in theta or delta frequency/rhythmic delta activity/periodic slow waves];
- Epileptiform abnormalities (EA) [sporadic epileptiform discharges/rhythmic or periodic epileptiform discharges/seizures/status epilepticus];
- Ictal-Interictal Continuum (IIC).

**Fig. 1:**
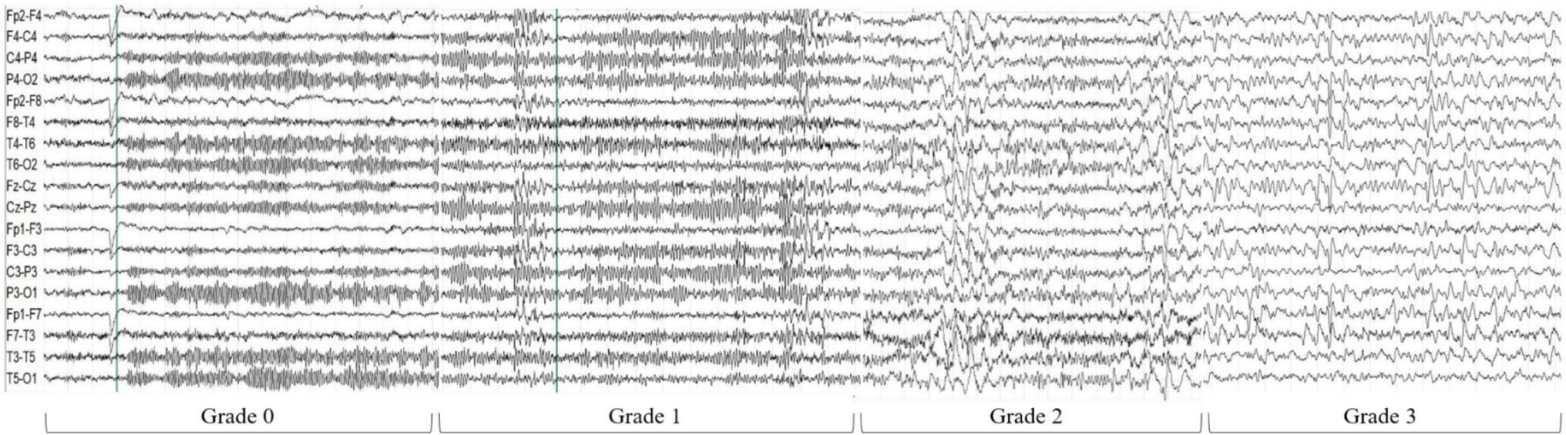
Examples of the EEG grading scale, *-Grade 0* normal EEG. *-Grade 1* normal background (alpha rhythm) with sporadic diffuse theta activity at moderate prevalence. *-Grade 2* predominant theta background frequency with sporadic diffuse delta activity at moderate prevalence. *-Grade 3* predominant delta background frequency with anterior-predominant diffuse sharp waves at high prevalence

The prevalence of the abnormalities (both NEA and EA) was defined as follows (correspondences with ACNS terminology in brackets):

- Low (corresponding to “occasional” and “rare”): < 1/min.
- Moderate (corresponding to “frequent”): ≥1/minute but less than 1 per 10 s.
- High (corresponding to “abundant”): ≥1 per 10 s.

In case of discrepancies, a definitive judgment was obtained by open debate among the two raters while keeping clinical outcomes hidden. After establishing a definitive description for each EEG recording, a grading scale (Table 1) was developed to assess its significance. Each recording was assigned a score ranging from 0 (normal) to 3 (severely abnormal). Figure 1 shows representative EEG pages for each grade.

**Table 1:** EEG grading scale

PBF	Theta activity	Delta activity^*^	State changes	Epileptiform abnormalities	Overall significance	Grade
Alfa/ Beta	+	Absent	Absent/ Physiological	Absent	Normal	0
Alfa/ Beta	++	+	Pathological	Absent	Slightly abnormal	1
Theta	+++	++	Not relevant	+	Moderately abnormal	2
Delta	Not relevant	+++	Not relevant	++/+++	Severely abnormal	3
Final Grade: 0 = Normal PBF ± Low prevalence Theta activity and/or physiological state changes. 1= Normal PBF + Moderate prevalence Theta activity and/or Low prevalence Delta activity and/or pathological state changes. 2= Theta PBF and/or Absence of reactivity and/or Moderate prevalence Delta activity and/or Low prevalence epileptiform abnormalities. 3= Delta PBF and/or High prevalence Delta activity and/or Moderate/High prevalence epileptiform abnormalities.						

*PBF* predominant background frequency, *+* low prevalence, *++* moderate prevalence, *+++* high prevalence, *NA* not applicable

^*^Comprehending both polymorphic and rhythmic delta activity

#### Quantitative evaluation

All the eligible records were segmented into artifact-free time windows, using Brain Vision Analyzer 2.2. This segmentation procedure, including the artifact detection, was carried out manually by one board-certified neurologist (FP). Hence, a final epoch representative of each EEG was obtained by assembling the time windows, ensuring that the minimum epoch duration was 20 s. Each epoch was then processed according to an *ad hoc* pipeline, written in Python [29], to extract quantitative features describing the characteristics of the signal both in the time and frequency domains.

At first, each epoch was processed by a comb filter to erase the powerline frequency and its multiple. Next, each epoch was segmented into overlapping time windows of duration *T *= 2.0 s, considering ∆*t *= 0.25 s as a time shift between consecutive windows. Such a segmentation made it possible to obtain multiple samples of the same feature from each epoch, increasing the accuracy of the final measures. No channels were excluded in this phase since the portions of signals associated with artifacts were removed manually.

Hence, the following features were computed for each epoch and window:

- The signal energy *Eω* for the frequency bands delta δ=[2, 4] Hz, theta θ=[4, 8] Hz, alpha α=[8, 14] Hz, and beta β=[14, 30] Hz; in particular, the energy associated with each band was obtained as the integral of the power spectral density (PSD) of the signal over the frequencies characterizing the target band and the PSD was estimated via the Fast Fourier Transform (FFT) algorithm.
- The signal entropy *H* and fractal dimension *F*, estimating the complexity of the signal in terms of amplitude and phase distribution; in particular, the fractal dimension was computed via the so-called Higuchi method.
- The phase transfer entropy *PTE* between the different EEG channels, denoting the influence that each channel exercises over the others; we highlighted that the PTE represents a causal connectivity metric and the computation of the PTE was not restricted to specific frequency ranges but considered the broadband signal, avoiding the possible biases due to band selection.
- Graph-based measures, including the in/out-degree, the betweenness and eigenvector centrality, the eccentricity of each EEG channel, and the diameter of the overall EEG signal; all these metrics were extracted from the directional graph representation of the signal that was obtained with the *PTE* as connectivity estimate, which indeed enables to represent both the magnitude and direction of channels’ relationship.

To appreciate the time-dependent variations in parameters and allow inter-patient comparison, the features concerning post-infusion EEGs were normalized with respect to the baseline EEG.

### EEGs selection for the analysis

To explore the possible role of EEG as predictive biomarker for ICANS we considered:

- Baseline EEGs: to determine whether pre-infusion EEG abnormalities might represent a risk factor for the development of ICANS.
- Post-infusion EEGs (T1; T3; T7; T14): to assess whether EEG changes precede and thus predict the clinical onset of neurotoxicity. To perform this analysis, we compared EEGs recorded prior to ICANS onset with those of patients not developing ICANS.

### Statistical analysis

In descriptive analysis, continuous variables have been summarized and presented through mean, standard deviation (SD) and range (min-max), categorical variables through absolute numbers (n) and percentages (%). For continuous variables the Mann-Whitney test was used to evaluate differences between two groups, while the Chi-square test was used for categorical variables. Associations between qualitative or quantitative assessments of EEG tracings (fixed and time-dependent variables) and the development of ICANS (outcome - time to event) have been assessed through univariable and multivariable Cox regression models. Time to enter in the analysis was the day of infusion. We performed two distinct analyses. In the first approach (“classical” Cox regression model), we used a fixed exposure, where baseline EEG features were considered as a constant predictor throughout the study period. In the second approach (“time-dependent” Cox regression model), we used a time-varying exposure model were the EEG values were updated at each time point (T1, T3, T7, T14), allowing for dynamic changes in EEG features over time. The role of any confounding factors (age, sex, clinical characteristics, therapy, etc.) has been considered in the models. The multivariable model was selected using a step-wise procedure using likelihood ratio test to select the best model. Results were presented with hazard ratios (HR) and 95% confidence intervals (95% CI). Cohen’s kappa was used to evaluate the inter-rater reliability between two raters (FB, PT) for the qualitative EEG evaluation. Statistical analysis was performed using Stata SE 14.2.

### Standard protocol approvals

The study was approved by our institutional review board, Ethical Committee AVEC of Bologna (protocol number: CE: 319/2021/Sper/AOUBo). Written informed consent was obtained from all enrolled patients, both for study participation and data publication. All procedures were conducted according to the latest version of the Declaration of Helsinki.

## Results

### Cohort baseline characteristics

A total of 68 consecutive patients (mean age 55.2±14 years, 20 females) affected by refractory/relapsed B-cell non-Hodgkin lymphoma underwent anti-CD19 CAR T-cell therapy from September 2019 to September 2022 and were included in the study. The baseline demographics, lymphoma characteristics and therapies are summarized in Supplementary Table 1.

### ICANS features

Neurotoxicity manifested in 22/68 (32.4%) patients, with an average onset time of 5.7 days (range 0–15) following the infusion. Among those affected, 9/22 (40.9%) patients experienced severe ICANS (grade ≥3), of whom 3 deceased early (two for fulminant cerebral edema and one for systemic complications). ICANS manifestations were consistently preceded by CRS (CRS features and its relationship with ICANS are described in Supplementary Table 2). Epileptic events (isolated tonic-clonic seizures and, in one case, non-convulsive status epilepticus) were observed in 3/22 patients, two of whom exhibited fulminant cerebral oedema. ICANS features are summarized in Supplementary Table 3.

### Qualitative EEG analysis

**Fig. 2:**
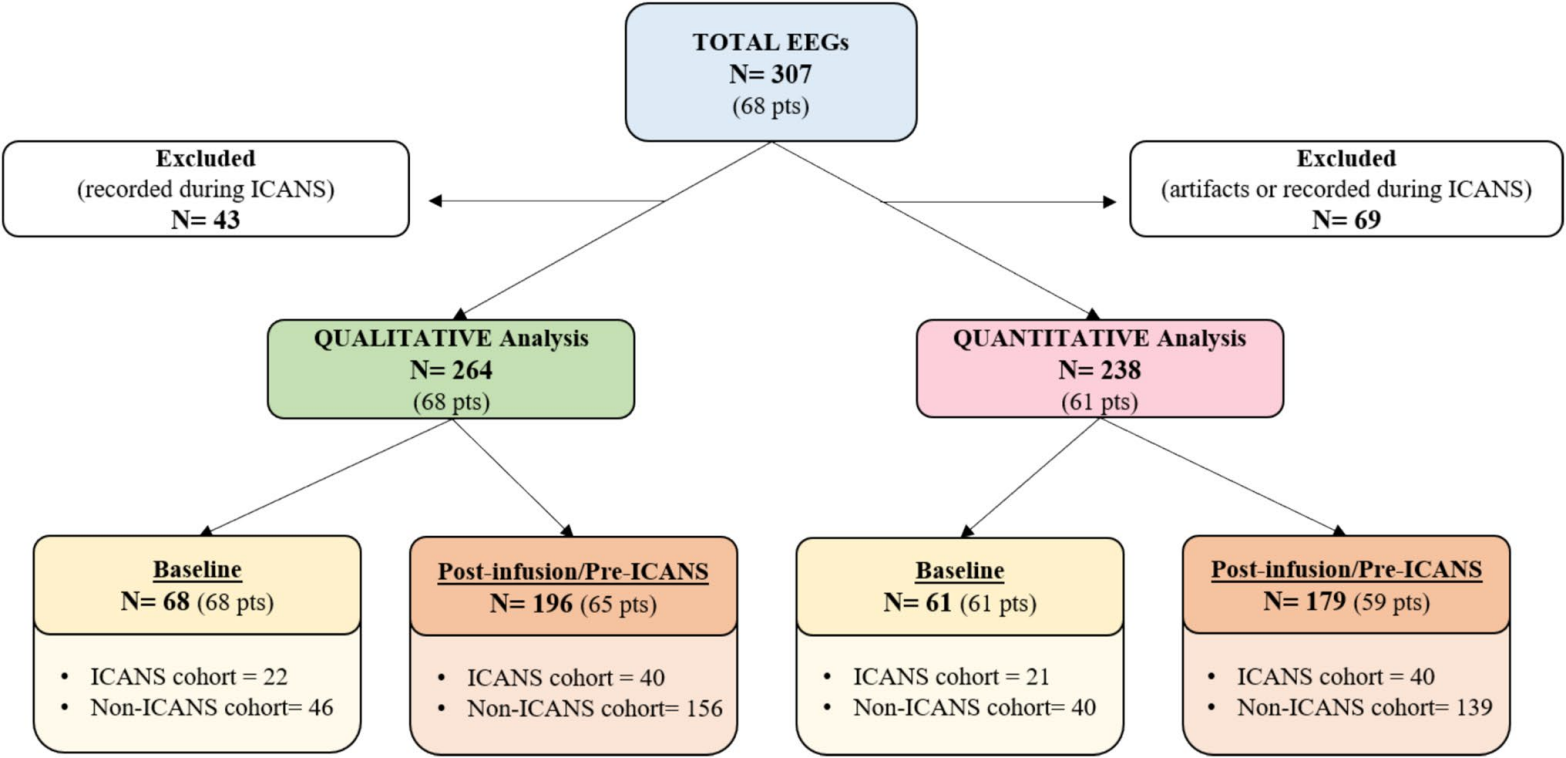
Flowchart of EEG records analysed qualitatively and quantitatively. *N* number of EEGs, *pts* patients

From the total pool, 43/307 (14.0%) EEGs were excluded since recorded during clinical manifestation of ICANS, thus 264/307 (86.0%) EEGs belonging to 68 patients were included in the final analysis. 68/264 (25.8%) EEGs were recorded in pre- infusion period and 196/264 (74.2%) after the infusion of CAR T-cells. Figure 2 describes EEGs distribution among the cohort, considering the time of recording.

The agreement between the two raters was strong (*k* = 0.88).

#### Baseline EEGs grading

Pathological findings were detected in 8/68 (11.8%) baseline EEGs. The grading score was 1 (slightly abnormal) in 6/8 (75%) and 2 (moderately abnormal) in 2/8 (25%) cases. The specific features of the pathological EEGs at baseline are shown in Supplementary Table 4.

#### Post-infusion EEGs grading

Among the 196 EEGs recorded after the infusion day up to T14, 156 (79.6%) belonged to non-ICANS cohort while 40 (20.4%) were from the ICANS cohort but recorded prior to its onset (pre-ICANS EEGs).

Pathological findings were observed in 12/156 (7.7%) records of the non-ICANS cohort and in 20/40 (50%) pre-ICANS EEGs. Grading score resulted 1 (slightly abnormal) in 10 non-ICANS vs 4 pre-ICANS, 2 (moderately abnormal) in 2 non-ICANS vs 11 pre-ICANS, 3 (severely abnormal) in 5 pre-ICANS vs none in the non-ICANS cohort.

Post-infusion EEG abnormalities anticipated ICANS onset by a median of 4 days (range: 1–9 days).

#### Risk of ICANS according to qualitative analysis

Risk of ICANS was significantly higher for both baseline EEGs showing grade 1 [HR 4.1; 95%CI 1.3–12.5] and grade 2 [HR 34.9; 95%CI 5.6–218.6] abnormalities (Table 2).

**Table 2:** ICANS risk according to qualitative EEGs analysis—univariable Cox regression models

Score	HR	[95% CI]	P-value
Baseline EEGs–fixed variable			
0	Ref	–	–
1	4.1	1.3–12.5	**0.013**
2	34.9	5.6–218.6	**<0.001**
1+2	5.8	2.6–12.9	**<0.001**
Post-infusion EEGs–time-dependent variable			
0	Ref	–	–
1	3.2	0.7–14.9	0.139
2	11.6	4.4–30.5	**<0.001**
3	9.7	2.6–36.6	**<0.001**

Bold refers the statistically significant values

*HR* Hazard Ratio; 95% *CI* 95% Confidence Interval

Taken together, baseline EEGs abnormalities [HR 5.8; 95%CI 2.6–12.9] were corroborated as independent risk factor for ICANS in multivariable analysis [HR 3.3; 95%CI 1.3–8.7] (Table 3).

**Table 3:** Risk factors for ICANS—univariable and multivariable Cox regression models

Feature vs reference category	Unadjusted HR (95% CI)	p-value	Adjusted HR (95% CI)	p-value
Age	1.0 (0.9–1.0)	0.217		
Sex				
Male vs female	4.0 (1.8–8.9)	**0.001**	3.7 (1.6–8.7)	**0.003**
Histology subtypes				
PMBCL vs DLBCL	4.7 (2.2–10.0)	**<0.001**		
MCL vs DLBCL	7.1 (3.5–14.2)	**<0.001**		
FL vs DLBCL	0.4 (0.0–3.0)	0.365		
Baseline EEG Abnormalities	5.6 (2.6–12.9)	**<0.001**	3.3 (1.3–8.7)	**0.014**
Ferritin				
>1000 vs <1000	2.0 (1.4–2.7)	**<0.001**	1.8 (1.3–2.6)	**0.001**
CAR T-cell Product				
Tisa-cell vs Axi-cell	0.4 (0.2–1.1)	0.074		
Brexu-cell vs Axi-cell	0.2 (0.3–1.4)	0.114		

Bold refers the statistically significant values

*HR* Hazard Ratio; 95% *CI* 95% Confidence Interval

Post-infusion EEGs abnormalities were associated to a significant higher risk of ICANS when scored 2 [HR 11.6; 95% CI 4.4–30.5] and 3 [HR 9.7; 95% CI 2.6–36.6], while grade 1 abnormalities showed a trend of higher risk [HR 3.2; 95%CI 0.7–14.9] (Table 2).

### Quantitative EEG analysis

From the total pool, 238/307 (77.5%) EEGs belonging to 61/68 patients (89.7%) were eligible for quantitative analysis (see Fig. 2), while 69 EEGs were excluded due to artifacts (*n* = 58) or because recorded during ICANS (*n *= 11).

No quantitative features at baseline were found to be significantly associated with a higher risk of ICANS development.

In post-infusion EEGs, energy values were significantly associated to the risk of ICANS development. Specifically, median theta energy [HR 1.10; 95%CI 1.03–1.16] and delta + theta/alfa ratio [HR 1.37; 95%CI 1.11–1.67] correlated to a higher risk of ICANS, while median beta energy emerged as protective factor [HR 0.91; 95%CI 0.85–0.97] (Table 4). On the contrary, signal complexity and connectivity measures were not associated to a higher risk of ICANS development.

**Table 4:** ICANS risk in post-infusion EEGs according to quantitative analysis-Energy (median values)—univariable Cox regression models

Feature	HR	[95% CI]	p-value
Beta energy	0.91	0.85–0.97	**0.006**
Alpha energy	0.97	0.94–1.01	0.160
Theta energy	1.10	1.03–1.16	**0.001**
Delta energy	1.04	0.99–1.11	0.091
Delta+ theta/alfa ratio	1.37	1.11–1.67	**0.002**

Bold refers the statistically significant values

*HR* Hazard Ratio; 95% *CI* 95% Confidence Interval

Cut-off values between tertiles were identified for theta energy and delta+ theta/alfa ratio. For both features, values above the cut-off between the second and the third tertile (2.2 and 0.31, respectively) were found to be significantly associated with a higher risk of developing ICANS (Supplementary Table 5).

### Differences in pre-infusion features between patients with and without baseline EEG abnormalities

Patients demonstrating abnormalities at baseline EEG were found to have significantly higher pre-infusion IL-6 (*p* = 0.031) and CRP (*p* = 0.014) serum levels. In contrast, demographics (age and sex), tumour-related factors (duration, disease status, stage, previous treatments), brain MRI abnormalities and neurological comorbidities did not differ among the two groups. The results of the post hoc analysis are available in Supplementary Table 6.

## Discussion

The present study investigates the role of EEG in evaluating patients undergoing CAR T-cell therapy, positioning it as a valuable predictive biomarker of ICANS both prior to and following CAR T-cell infusion. We rigorously assessed a large dataset of EEG recordings from CAR T-cell therapy recipients, employing a protocol with predetermined evaluations for all subjects and blinded analysis by highly specialized experts. While interrater variability typically poses a problem in the interpretation of EEGs [30], the use of an ad hoc rating scale designed to highlight gross differences allowed us to achieve strong agreement between raters. Additionally, we expanded the available data on quantitative EEGs analysis in CAR-T therapy recipients, which is currently limited [21].

Our findings underscore a dual role of EEG in assessing ICANS risk: firstly, by aiding in the identification of susceptible patients at baseline, and secondly, by predicting the clinical onset of ICANS after CAR T-cell infusion.

### EEG as a screening tool

Patients with pre-infusion EEG abnormalities of any grade had a six times higher risk of developing ICANS compared to those with a normal EEG, a finding confirmed in the multivariable analysis with confounding factors, supporting our preliminary findings [24] and the recent study from Hernani et al. [25]

The precise significance of baseline EEG abnormalities is challenging to ascertain and requires to be investigated within the context of the pre-infusion period and intrinsic individual characteristics. Notably, no significant neurological comorbidities nor CNS lymphoma involvement were present in our cohort. Instead, patients with baseline EEG abnormalities had higher serum IL-6 and CRP levels compared to those with normal EEG, in line with previous reports [12] suggesting that a pre-infusion inflammatory state may predispose to the development of ICANS. In this respect, bridge therapies (i.e. chemotherapy and immunotherapy) may act exacerbating the hyperinflammatory condition [31]. The pre-infusion inflammatory state may facilitate Blood-Brain Barrier (BBB) damage, making the central nervous system more vulnerable to cytokine-mediated neuroinflammation secondary to CRS, which consistently anticipated ICANS in our cohort. Interestingly, pre-operative EEG abnormalities and elevated inflammatory markers may predict post-operative delirium [32, 33], a condition with a multifactorial pathogenesis that shares neuroinflammation with ICANS [34]. This could support the hypothesis that EEG abnormalities may reflect a non-specific marker of cerebral vulnerability and predisposition to cytokine storm-associated encephalopathies [34].

Visual (qualitative) EEGs evaluation proved to be highly effective in assessing the risk of ICANS at baseline outperforming quantitative analysis which, conversely, yielded non-significant results. Potentially contributing factors include the bias related to the selection of artifact-free segments in quantitative analysis, and the fact that most altered pre-infusion EEGs had sporadic slowing within a normal background activity, making the detection of abnormalities through quantitative analysis challenging. Thus, visual analysis of baseline EEG, a widely available technique in all neurological centers involved in CAR-T therapy, could truly represent a crucial tool for screening candidates at risk of developing ICANS, also considering the increasing evidence of effective prophylactic protocols [13, 14].

Significantly reducing the incidence and severity of ICANS could have transformative implications. Currently, CAR T-cell therapy demands extensive resources, necessitating inpatient delivery and close outpatient monitoring due to the risk of severe toxicities, which may be mitigated by implementing a prophylactic strategy [13]. The employment of EEG as predictive biomarkers for ICANS during the pre-infusion assessment could guide patient selection for randomized trials on prophylactic agents.

### EEG as a predictive tool

Our study provides compelling evidence that abnormalities in post-infusion EEG can precede and potentially predict the clinical onset of ICANS. Although ICANS is defined clinically, our findings suggest that EEG changes consistent with encephalopathy may emerge in the presymptomatic stage, anticipating clinical onset by a median of 4 days.

Both visual and quantitative assessments were able to detect EEG changes, with the former demonstrating superior performance. Nevertheless, quantitative analysis could prove beneficial in situations where continuous EEG monitoring is accessible, potentially allowing for automated detection of background slowing and subsequently triggering alerts for physicians.

These findings suggest that the incorporation of daily EEGs in neurological monitoring post-CAR T-cell infusion could facilitate early detection of ICANS onset.

Given the ease of application of EEG and the availability of effective therapies for ICANS, this protocol adjustment offers a significantly advantageous cost-to-benefit ratio. Indeed, even if ICANS is potentially fatal and characterized by a relevant morbidity, it can be controlled by immunomodulating treatments especially if promptly initiated [12].

### Study limitations

The monocentric design of the study constrains the generalizability of our findings. However, it concurrently streamlined coordination among researchers and yielded a homogeneous sample, thereby minimizing external confounding variables.

Furthermore, while the expertise of the evaluators and their blindness to clinical data ensure the accuracy of the assessments, it may also limit the generalizability of the findings to settings with less specialized resources or expertise.

The EEG Grading Scale we have developed is easily applicable since the different grades are readily distinguishable based on the background activity’s frequency band and the identification of gross EEG abnormalities, and might therefore be easily applicable even by neurologists with basic EEG training. However, we the scale needs to be validated on a larger cohort of patients.

Given the limited sample size, we lacked sufficient power to conduct a reliable ordinal analysis stratified by ICANS severity grade; future studies with a larger cohort may allow to see if specific EEG features correlate with more severe ICANS.

## Conclusions

Our study establishes EEG as a pivotal tool for evaluating patients undergoing CAR T-cell therapy enhancing the ability to identify patients susceptible to ICANS who could benefit from prophylactic treatment and, after the infusion, anticipating ICANS onset and allowing for timely interventions that are essential to improve patients’ outcome. EEG emerges as an optimal predictive biomarker for ICANS owing to its widespread accessibility, simplicity of execution, prompt result acquisition, and distinctive ability to offer varied information beyond clinical assessment and laboratory investigations. Although further prospective studies are necessary to validate our findings, incorporating EEG monitoring into the clinical protocols for CAR T-cell therapy candidates and recipients already appears to be reasonable.

## Supplementary Information

Below is the link to the electronic supplementary material.

Supplementary file1 (DOCX 1247 KB)

## References

[1] Sterner RC, Sterner RM (2021) CAR-T cell therapy: current limitations and potential strategies. Blood Cancer J 11:69 33824268 10.1038/s41408-021-00459-7 PMC8024391

[2] Lee DW et al (2019) ASTCT consensus grading for cytokine release syndrome and neurologic toxicity associated with immune effector cells. Biol Blood Marrow Transplant 25:625–638 30592986 10.1016/j.bbmt.2018.12.758 PMC12180426

[3] Grant SJ et al (2022) Clinical presentation, risk factors, and outcomes of immune effector cell-associated neurotoxicity syndrome following CAR-T cell therapy: a systematic review. Transplant Cell Ther 28:294–302 35288347 10.1016/j.jtct.2022.03.006 PMC9197870

[4] Gust J et al (2017) Endothelial activation and blood-brain barrier disruption in neurotoxicity after adoptive immunotherapy with CD19 CAR-T cells. Cancer Discov 7:1404–1419 29025771 10.1158/2159-8290.CD-17-0698 PMC5718945

[5] Morris EC, Neelapu SS, Giavridis T, Sadelain M (2022) Cytokine release syndrome and associated neurotoxicity in cancer immunotherapy. Nat Rev Immunol 22:85–96 34002066 10.1038/s41577-021-00547-6 PMC8127450

[6] Rubin DB et al (2020) Clinical predictors of neurotoxicity after chimeric antigen receptor T-cell therapy. JAMA Neurol 77:1536 32777012 10.1001/jamaneurol.2020.2703 PMC7418044

[7] Bachy E, Le Gouill S, Di Blasi R et al (2022) A real-world comparison of tisagenlecleucel and axicabtagene ciloleucel CAR T cells in relapsed or refractory diffuse large B cell lymphoma. Nat Med 28(10):2145–2154. 10.1038/s41591-022-01969-y 36138152 10.1038/s41591-022-01969-y PMC9556323

[8] Siddiqi T et al (2017) Patient characteristics and pre-infusion biomarkers of inflammation correlate with clinical outcomes after treatment with the defined composition, CD19-targeted CAR T cell product, JCAR017. Blood 130:193

[9] Karschnia P et al (2019) Clinical presentation, management, and biomarkers of neurotoxicity after adoptive immunotherapy with CAR T cells. Blood 133:2212–2221 30808634 10.1182/blood-2018-12-893396

[10] Belin C et al (2020) Description of neurotoxicity in a series of patients treated with CAR T-cell therapy. Sci Rep 10:18997 33149178 10.1038/s41598-020-76055-9 PMC7642402

[11] Butt OH, Zhou AY, Ances BM, DiPersio JF, Ghobadi A (2023) A systematic framework for predictive biomarkers in immune effector cell-associated neurotoxicity syndrome. Front Neurol 14:1110647. 10.3389/fneur.2023.1110647 36860569 10.3389/fneur.2023.1110647 PMC9969296

[12] Neelapu SS, Tummala S, Kebriaei P et al (2018) Chimeric antigen receptor T-cell therapy—assessment and management of toxicities. Nat Rev Clin Oncol 15:47–56 28925994 10.1038/nrclinonc.2017.148 PMC6733403

[13] Strati P et al (2023) A phase 1 study of prophylactic anakinra to mitigate ICANS in patients with large B-cell lymphoma. Blood Adv 7(21):6785–6789 37389847 10.1182/bloodadvances.2023010653 PMC10692290

[14] Park JH, Nath K, Devlin SM et al (2023) CD19 CAR T-cell therapy and prophylactic anakinra in relapsed or refractory lymphoma: phase 2 trial interim results. Nat Med 29:1710–1717. 10.1038/s41591-023-02404-6 37400640 10.1038/s41591-023-02404-6 PMC11462637

[15] Ponten SC, Tewarie P, Slooter AJC, Stam CJ, Van Dellen E (2013) Neural network modeling of EEG patterns in encephalopathy. J Clin Neurophysiol 30:545–552 24084188 10.1097/WNP.0b013e3182a73e16

[16] Herlopian A, Dietrich J, Abramson JS, Cole AJ, Westover MB (2018) EEG findings in CAR T-cell therapy-related encephalopathy. Neurology 91:227–229 29959264 10.1212/WNL.0000000000005910 PMC6093761

[17] Beuchat I et al (2022) EEG findings in CAR T-cell-associated neurotoxicity: clinical and radiological correlations. Neuro Oncol 24:313–325 34265061 10.1093/neuonc/noab174 PMC8804895

[18] Huby S et al (2023) Frontal intermittent rhythmic delta activity is a useful diagnostic tool of neurotoxicity after CAR T-cell infusion. Neurol Neuroimmunol Neuroinflamm 10:e200111 37059470 10.1212/NXI.0000000000200111 PMC10119810

[19] Gust J, Annesley CE, Gardner RA, Bozarth X (2021) EEG correlates of delirium in children and young adults with CD19-directed CAR T cell treatment-related neurotoxicity. J Clin Neurophysiol 38:135–142 31851018 10.1097/WNP.0000000000000669 PMC7292745

[20] Sokolov E et al (2020) Language dysfunction-associated EEG findings in patients with CAR-T related neurotoxicity. BMJ Neurol Open 2:e000054 33681787 10.1136/bmjno-2020-000054 PMC7871716

[21] Eckhardt CA et al (2023) Automated detection of immune effector cell-associated neurotoxicity syndrome via quantitative EEG. Ann Clin Transl Neurol. 10.1002/acn3.51866 37545104 10.1002/acn3.51866 PMC10578889

[22] Jones DK et al (2022) EEG-based grading of immune effector cell-associated neurotoxicity syndrome. Sci Rep 12:20011 36414694 10.1038/s41598-022-24010-1 PMC9681864

[23] Satyanarayan S et al (2023) Continuous EEG monitoring detects nonconvulsive seizure and Ictal-Interictal continuum abnormalities in moderate to severe ICANS following systemic CAR-T therapy. Neurohospitalist 13:53–60 36531846 10.1177/19418744221128852 PMC9755619

[24] Pensato U, Amore G, Muccioli L, Sammali S, Rondelli F, Rinaldi R et al (2023) CAR t-cell therapy in BOlogNa–NEUrotoxicity TReatment and assessment in lymphoma (CARBON–NEUTRAL): proposed protocol and results from an Italian study. J Neurol 270:2659–2673. 10.1007/s00415-023-11595-4 36869888 10.1007/s00415-023-11595-4

[25] Hernani R, Aiko M, Victorio R et al (2024) EEG before chimeric antigen receptor T-cell therapy and early after onset of immune effector cell-associated neurotoxicity syndrome. Clin Neurophysiol 163:132–142. 10.1016/j.clinph.2024.04.014 38733703 10.1016/j.clinph.2024.04.014

[26] Pensato U, Amore G, D’Angelo R et al (2022) Frontal predominant encephalopathy with early paligraphia as a distinctive signature of CAR T-cell therapy-related neurotoxicity. J Neurol 269(2):609–615. 10.1007/s00415-021-10766-5 34424399 10.1007/s00415-021-10766-5 PMC8381707

[27] von Elm E, Altman DG, Egger M, Pocock SJ, Gøtzsche PC, Vandenbroucke JP (2007) The Strengthening the reporting of observational studies in epidemiology (STROBE) statement:guidelines for reporting observational studies. Lancet 370:1453–1457 18064739 10.1016/S0140-6736(07)61602-X

[28] Hirsch LJ et al (2021) American clinical neurophysiology society’s standardized critical care EEG terminology: 2021 version. J Clin Neurophysiol 38:1–29 33475321 10.1097/WNP.0000000000000806 PMC8135051

[29] van Rossum G (1995) Python tutorial, technical report CS-R9526. Centrum voor Wiskunde en Informatica (CWI), Amsterdam

[30] Williams GW, Lüders HO, Brickner A, Goormastic M, Klass DW (1985) Interobserver variability in EEG interpretation. Neurology 35(12):1714–1719. 10.1212/wnl.35.12.1714 4069362 10.1212/wnl.35.12.1714

[31] Edwardson DW, Parissenti AM, Kovala AT (2019) Chemotherapy and inflammatory cytokine signalling in cancer cells and the tumour microenvironment. In: Ahmad A (ed) Breast cancer metastasis and drug resistance. advances in experimental medicine and biology vol 1152. Springer, Cham. 10.1007/978-3-030-20301-6_9 10.1007/978-3-030-20301-6_9 31456184

[32] Bruzzone M, Walker J, Chapin B et al (2023) The role of the perioperative use of EEG as a predictor/diagnostic tool for post-operative delirium: systematic review (P10–1008). Neurology 100(10_supplement_2):4678. 10.1212/WNL.0000000000204149

[33] Capri M, Yani SL, Chattat R et al (2014) Pre-operative, high-IL-6 blood level is a risk factor of post-operative delirium onset in old patients. Front Endocrinol (Lausanne) 5:173. 10.3389/fendo.2014.00173 25368603 10.3389/fendo.2014.00173 PMC4201145

[34] Pensato U, Muccioli L, Cani I et al (2021) Brain dysfunction in COVID-19 and CAR-T therapy: cytokine storm-associated encephalopathy. Ann Clin Transl Neurol 8(4):968–979. 10.1002/acn3.51348 33780166 10.1002/acn3.51348 PMC8045903

