# Supplementary material for: EEG as a predictive biomarker of neurotoxicity in anti-CD19 CAR T-cell therapy
Source: J Neurol. 2025 Apr 25;272(5):360. doi: 10.1007/s00415-025-13102-3 (PMC12031834; doi:10.1007/s00415-025-13102-3)
Supplement: Supplementary file 1 — Supplementary file1 (DOCX 1247 KB) [file 415_2025_13102_MOESM1_ESM.docx]

**SUPPLEMENTS**

**
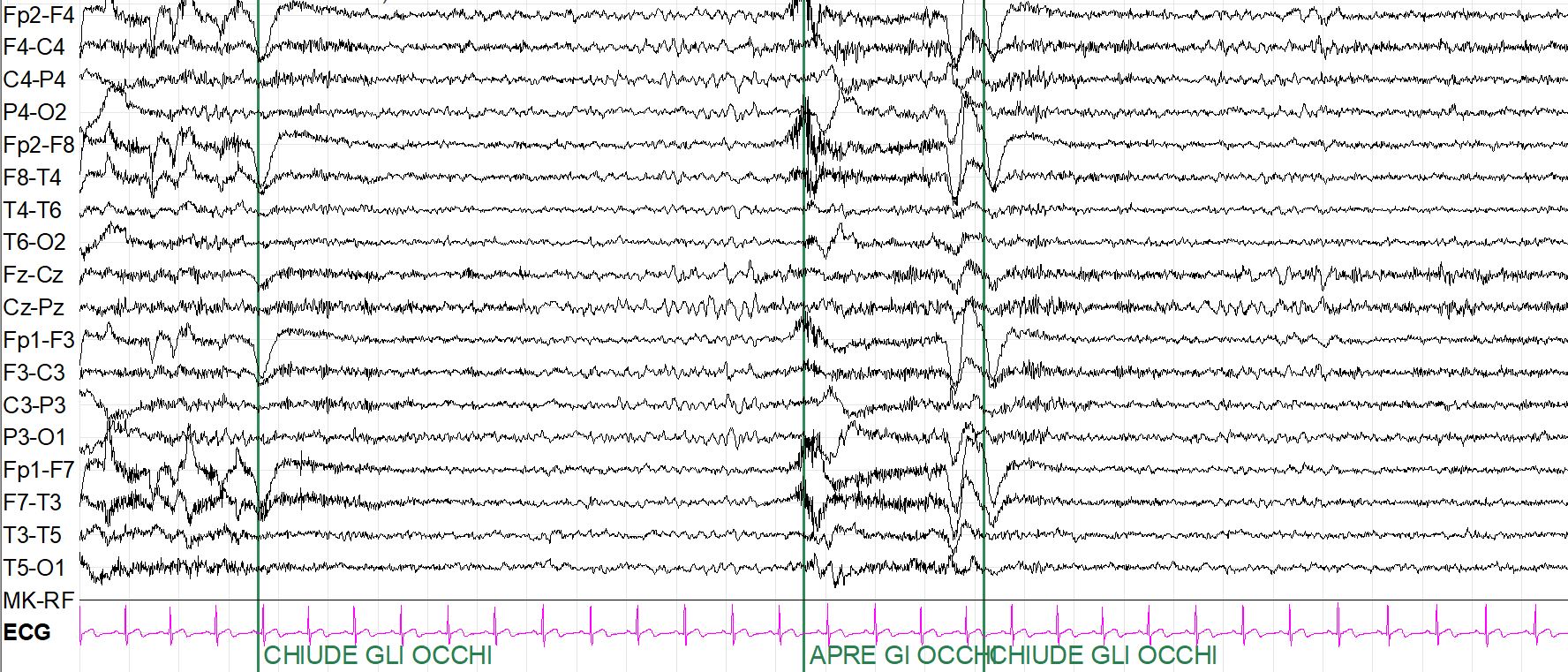

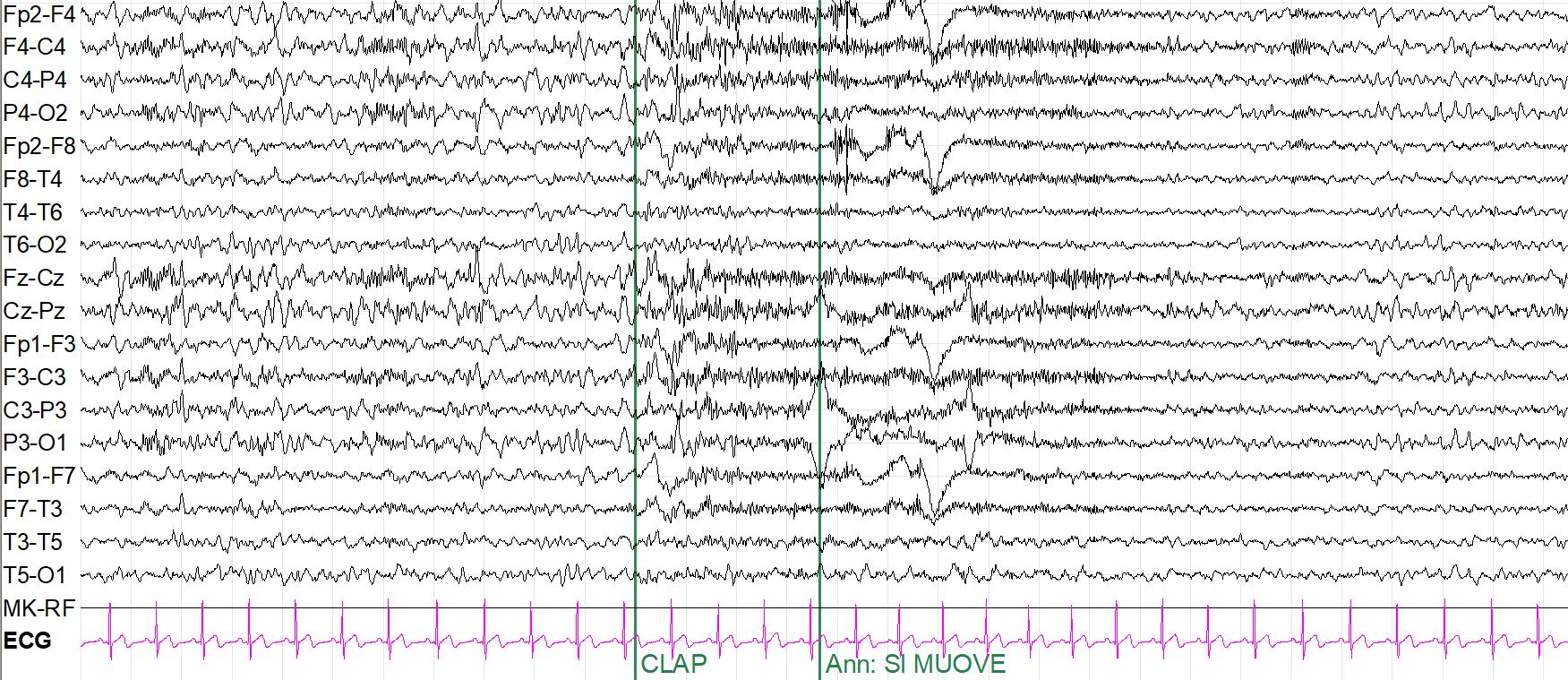
Supplementary Figure 1: Example of pathological State Changes**

Figure: posterior alpha rhythm replaced by long-lasting diffuse delta activity, rapidly emerging after eye-closure**
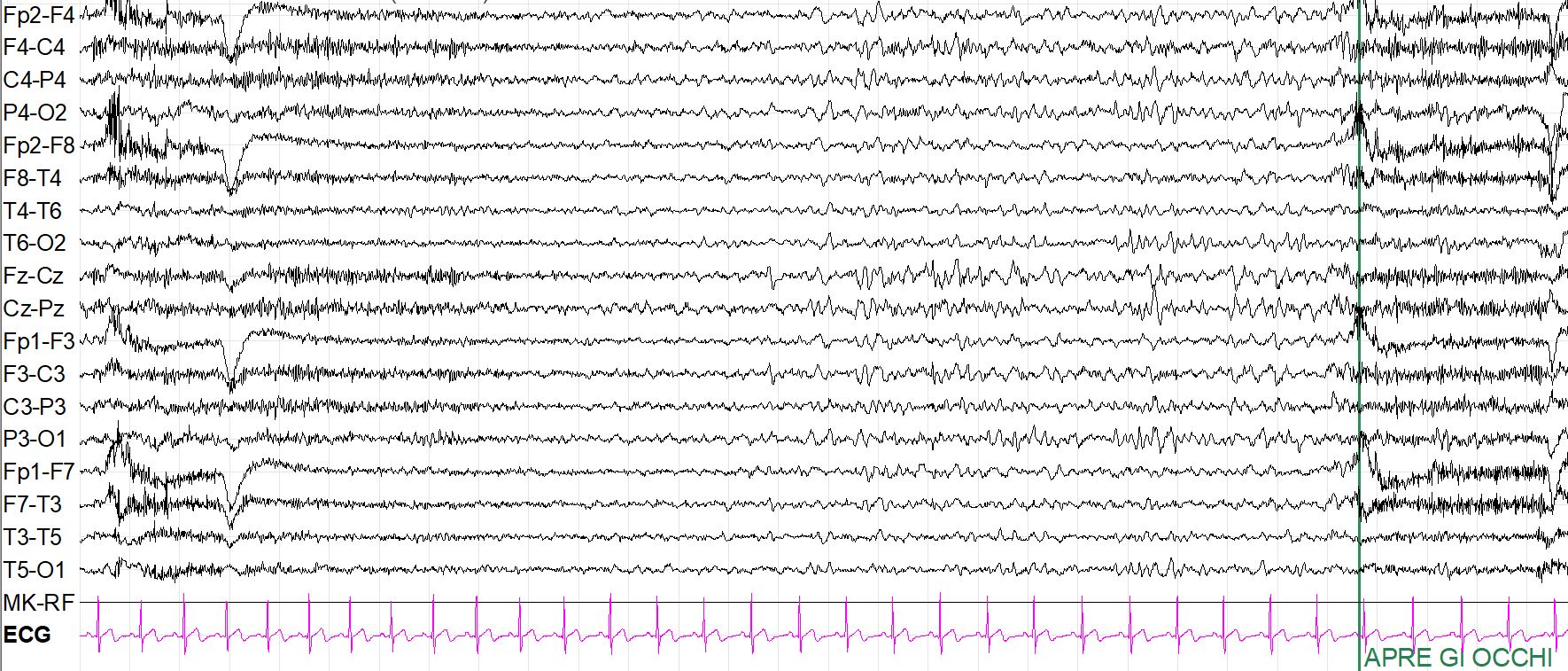
**. Typical graphoelements of NREM sleep phase 2 (spindles, vertex waves) are visible.

**Supplementary Table 1: Baseline characteristics of the cohort**

|  | Total patients  (n = 68) |
| --- | --- |
|  |  |
| Age (years)  Mean, (SD) [range] | 55.2 (±14) [19-70] |
| Female sex | 20 (29.4%) |
| Histology  DLBCL  PMBCL  MCL  FL | 50 (73.5%)  8 (11.8%)  9 (13.2%)  1 (1.5%) |
| Bulky (>7cm) | 32 (47.1%) |
| Disease status  Early refractory  Refractory  Relapsed | 56 (82.4%)  4 (5.9%)  10 (14.7%) |
| Bridge therapy  Pembrolizumab  Steroids  Chemotherapy  Radiotherapy  Combinations | 48 (70.6%)  6 (8.8%)  6 (8.8%)  25 (36.8%)  4 (5.9%)  7 (10.3%) |
| Previous treatments (n)  Mean, (SD) [range] | 2.9 (±1.3) [1-8] |
| ASCT | 23 (33.8%) |
| History of lymphoma (months)  Mean, (SD) [range] | 51.1 (±60.2) [5-264] |
| Disease stage III-IV | 52 (76.5%) |
| Serum inflammatory markers  IL-6 (pg/mL)  IL-10 (pg/mL)  IL-8 (pg/mL)  Ferritin (ng/mL)  CRP (mg/dL)  D-dimer (mg/L) | 17.5 (±28.6) [0.2-7.6]  9.5 (±27.2) [0-185]  45 (±75.8) [5-574]  564 (±1139.1) [11-6787]  2.44 (±4.5) [0.1-19.2]  1.7 (±2.9) [0.2-7.3] |
| CAR T-cells product  Axi-cel  Tisa-cel  Brexu-cel | 31 (45.6%)  28 (41.2%)  9 (13.2%) |

LEGEND: ASCT= autologous stem cell transplantation; DLBCL= Diffuse large B-cell lymphoma; PMBCL= Primary mediastinal B-cell lymphoma; MCL= Mantle cell lymphoma, FL= Follicular lymphoma.

**Supplementary Table 2: Associations between CRS-related variables and ICANS**

|  | **Total patients**  **(n = 68)** | **ICANS** | | **p-value** |
| --- | --- | --- | --- | --- |
|  |  | **YES (n= 22)** | **NO (n=46)** |  |
| **CRS**  Onset (T)  Severe CRS (≥3)  Duration (days) | 58/68 (85.3%)  2.4 [0-11]  6/58 (10.3%)  5.8 [1-30] | 22/22 (100%)  1.2 [0-4]  6/22 (27.3%)  7.7 [2-30] | 36/46 (78.3%)  3.2 [0-11]  0/36 (0%)  4.75 [1-2] | **0.001**  **<0.001**  **<0.001** |
| **Tocilizumab (n)**  (mean, SD) [range] | 1.6 (±1.4) [0-4] | 2.4 (±1.1) [0-4] | 1.3 (±1.3) [0-3] | **0.002** |
| **Serum Inflammatory Markers Peaks** (mean, SD) [range]  IL-6 (pg/mL)  IL-10 (pg/mL)  IL-8 (pg/mL) | 1511.5 (±1841.1)  [4.7-6773]  145.7 (±681.1)  [1-5406]  429.3 (±710.7)  [31-5000] | 2722.4 (±2093.3)  [59-6773]  405 (±1193.9)  [4-5406]  730.7 (±1085.7)  [43-5000] | 883.1 (±1338.4)  [4.7-4899]  24.7 (±29.4)  [1-115]  288.7 (±397.7)  [31-2446] | **<0.001**  **<0.001**  **0.011** |
| Ferritin (ng/mL) | 1771.4 (±2353.7)  [22-7500] | 3589.4 (±3101.8)  [155-7500] | 902 (±1222)  [22-7500] | **0.002** |
| CRP (mg/dL)  D-dimer (mg/L) | 12.8 (±10.0)  [0.2-46.4]  4.3 (±3.8)  [0.2-7.2] | 18.5 (±11.1)  [3.8-46.4]  6.9 (±4.7)  [0.4-7.2] | 8.8 (±7.4)  [0.2-30.9]  2.3 (±1.8)  [0.2-6.6] | **<0.001**  **<0.001** |

**Supplementary Table 3. Features of the ICANS cohort**

| **ICANS patients (n)** | 22/68 (32.4%) | | |
| --- | --- | --- | --- |
| **Disease timing and grading** | | |  |
| Severe ICANS (**≥**3) | | | 9/22 (40.9%) |
| Onset (T) (SD) [range] | | | 5.5 (±3.7) [0-15] |
| Mean duration (days) (SD) [range] | | | 10 (±12.2) [1-52] |
| **Relationship with CRS** | | |  |
| CRS prior or concomitant to ICANS | | 22/22 (100%) | |
| Mean time from CRS onset to ICANS onset (days) (SD) [range] | | 4.3 (±3.5) [0-14] | |
| **ICU**  ICU admission  Length of stay (days) (SD) [range] | | 11/22 (50.0%)  9.7 (± 6.9) [2-24] | |
| **Clinical features** | | |  |
| Seizures  NCSE  Frontal lobe dysfunction  Ideo-motor slowing  Coma  Dysgraphia/palygraphia | | | 2/22 (9.1%)  1/22 (4.5%)  16/22 (72.7%)  20/22 (90.9%)  9/22 (40.9%)  9/22 (40.9%) |
| **Neuroimaging findings**  Unremarkable | | | 12/19 (63.2%) |
| Focal or diffuse cerebral edema | | | 5/19 (26.3%) |
| Leptomeningeal enhancement | | | 1/19 (5.2%) |
| Intracerebral hemorrhage | | | 1/19 (5.2%) |
| **Treatment**  None | | | 2/22 (9.1%) |
| Steroids | | | 10 (45.5%) |
| Anakinra | | | 7 (31.8%) |
| Siltuximab | | | 8 (36.4%) |
| **Early neurotoxicity outcome** | | |  |
| Spontaneous resolution | | | 2 (9.1%) |
| Resolution following immunotherapy | | | 17 (77.3%) |
| Neurotoxicity-related death  Systemic complications-related death | | | 2 (9.1%)  1 (4.5%) |

Legend: ICU= Intensive Care Unit; NCSE= Non-Convulsive Status Epilepticus; SD= Standard deviation; T= days from the infusion.

**Supplementary Table 4: Features of the pathological baseline EEGs**

| Patient | PBF | Reactivity | Sporadic Theta | SporadicDelta | State changes | EA | Significance | Score | ICANS |
| --- | --- | --- | --- | --- | --- | --- | --- | --- | --- |
| 1.AA | Alpha | Present | ++ | + | - | - | Slightly abnormal | **1** | Yes |
| 2.EB | Alpha | Present | ++ | + | Physio | - | Slightly abnormal | **1** | Yes |
| 3.AB | Theta | Present | +++ | ++ | - | - | Moderately abnormal | **2** | Yes |
| 4.CD | Alpha | Present | - | - | Patho  logical | - | Slightly abnormal | **1** | Yes |
| 5.GI | Alpha | Present | - | - | Patho  logical | - | Slightly abnormal | **1** | Yes |
| 6.IM | Theta | Present | +++ | ++ | - | - | Moderately abnormal | **2** | Yes |
| 7.MM | Alpha | Present | - | - | Patho  logical | - | Slightly abnormal | **1** | No |
| 8.FR | Alpha | Present | ++ | + | - | - | Slightly abnormal | **1** | Yes |

Legend: PBF= predominant background frequency; EA= epileptiform abnormalities; - = absent; + = low prevalence; ++ = moderate prevalence; +++ = high prevalence.

**Supplementary Table 5: Cut off values-Energy (time-dependent variables) in association of ICANS - univariable Cox regression models**

| Feature | HR | [95% CI] | P-value | Cut off |
| --- | --- | --- | --- | --- |
| **Theta energy**  First tertile    Second tertile  Third tertile | Ref  0.4  3.5 | -  0.7-2.2  1.1-10.9 | -  0.282  **0.029** | ***-****0.56*  ***2.2*** |
| **Delta + theta/alfa**  First tertile    Second tertile  Third tertile | Ref  2.0  4.1 | -  0.4-10.4  0.9-18.5 | -  0.406  **0.068** | -*0.16*  ***0.31*** |

Legend: HR = Hazard Ratio; 95% CI = 95% Confidence Interval

**Supplementary Table 6: Differences in pre-infusion features between patients with and without baseline EEG abnormalities.**

|  | **Baseline EEG abnormalities** | | **p-value** |
| --- | --- | --- | --- |
|  | **YES (n= 8)** | **NO (n=60)** |  |
| **Age** (years) (mean; range) | 54.4 (19-68) | 55.4 (21-70) | 0.848 |
| **Female sex** | 4/8 (50%) | 8/60 (13.3%) | 0.174 |
| **Histology**  DLBCL  PMBCL  MCL  FL | 5/8 (62.5%)  2/8 (25%)  0/8 (0%)  1/8 (12.5%) | 45/60 (75%)  6/60 (10%)  9/60 (15%)  0/60 (0%) | **0.017** |
| **Bulky** (>7cm) | 4/8 (50%) | 28/60 (46.7%) | 0.859 |
| **Disease status**  Early refractory (<6 months)  Refractory (6-12 months)  Relapsed (>12 months) | 7/8 (87.5%)  1/8 (12.5%)  0/8 (0%) | 49/60 (81.7%)  3/60 (5%)  8/60 (13.3%) | 0.413 |
| **Bridge therapy**  Pembrolizumab  Steroids  Chemotherapy  Radiotherapy  Combinations | 7/8 (87.5%)  1/7 (14.3%)  2/7 (28.6%)  2/7 (28.6%)  0/7 (0%)  2/7 (28.6%) | 41/60 (68.3%)  5/41 (12.2%)  4/41 (9.8%)  23/41 (56.1%)  4/41 (9.8%)  5/41 (12.2%) | 0.054 |
| **Previous treatments** (n) (mean, range) | 2.5 (2-3) | 3 (1-8) | 0.487 |
| **ASCT** | 4/8 (50%) | 19/60 (31.7%) | 0.303 |
| **History of lymphoma** (months) (mean, range) | 19.6 (9-36) | 55.3 (5-264) | 0.826 |
| **Disease stage** III-IV | 7/8 (87.5%) | 45/60 (75%) | 0.434 |
| **RMN abnormalities** | 6/8 (75%) | 34/60 (56.7%) | 0.347 |
| **Pre-infusion serum inflammatory markers**  IL-6 (pg/mL)  IL-10 (pg/mL)  IL-8 (pg/mL)  Ferritin (ng/mL)  CRP (mg/dL)  D-dimer (mg/L) | 36 (3.2-150)  1.3 (0-4)  38.3 (10-121)  1473.4 (11-6787)  7.7 (0.1-19.2)  1.8 (0.19-7.32) | 14.5 (0.5-129)  10.3 (0-185)  45.9 (5-574)  442 (11-6534)  1.6 (0.1-18.5)  1.8 (0.2-6.3) | **0.031**  0.344  0.956  0.202  **0.014**  0.195 |
